# Potential predictors of delay in initial treatment contact after the first onset of depression in Japan: a clinical sample study

**DOI:** 10.1186/1752-4458-8-50

**Published:** 2014-12-05

**Authors:** Yoshiyo Oguchi, Atsuo Nakagawa, Mitsuhiro Sado, Dai Mitsuda, Yuko Nakagawa, Noriko Kato, Sayuri Takechi, Mitsunori Hiyama, Masaru Mimura

**Affiliations:** Department of Neuropsychiatry, Keio University School of Medicine, 35 Shinanomachi, Shinjuku-ku, Tokyo, 160-8582 Japan; Center for Clinical Research, Keio University School of Medicine, Tokyo, Japan; Department of Psychiatry, National Hospital Organization, Tokyo Medical Center, Tokyo, Japan

**Keywords:** Duration of untreated illness, Help-seeking, Major depressive disorder, Marital status

## Abstract

**Background:**

A growing body of evidence shows that reducing the duration of untreated illness (DUI) correlates with improved clinical outcome and course of depression. However, the factors involved in delaying treatment contact after the first onset of lifetime depression are not fully understood. This cross-sectional study aims to identify the characteristics that may predict the delay in initial treatment contact after the first onset of lifetime depression by comparing the socio-demographics and clinical characteristics between those with longer and shorter DUI in a well-characterized Japanese clinical sample.

**Methods:**

Ninety-five patients with depression with longer (>12 months) and shorter DUI (≤12 months) at three Japanese outpatient clinics were studied. Subjects received a comprehensive evaluation, including semi-structured clinical interviews and assessment battery, and their clinical charts were reviewed.

**Results:**

Of the total sample, the median of DUI was 4 months (interquartile range (IQR) 25th–75th percentile, 2–13). We found that 72.6% of patients seek treatment contact within the first year of depression onset. Multivariate logistic regression analysis showed that longer DUI in patients was associated with marital status (never married). Further, the DSM-IV melancholic features approached significance.

**Conclusions:**

Our findings suggest that most Japanese patients with depression are likely to seek treatment within 1 year of onset, and that marital status and melancholia may be potential predictors of the delay in the initial treatment contact after the first onset of lifetime depression.

## Background

Major depression is a common medical disorder associated with marked functional impairment [1, 2], and is recognized as one of the leading causes of disability in a population [3]. This has a significant impact on individuals and society, and is predicted to be the second largest contributor to the global disease burden by 2020 among high-income countries [4]. Therefore, providing effective and timely treatment is crucial for this debilitating disorder.

Relevant treatment guidelines recommend antidepressant pharmacotherapy, evidence-based psychotherapy, or a combination of the two treatments as an initial treatment option for acute depression [5–7]. However, published reports have shown that a large proportion of individuals with depression, delay and fail to establish initial treatment contact [8–10], thereby translating the individual’s current distress into more impervious and refractory conditions [11–13]. In fact, there is a growing body of evidence showing that reducing the duration of untreated illness (DUI), defined as the interval between the onset of a patient’s first psychiatric episode and the beginning of the first appropriate treatment [14], correlates with improved clinical outcome and the course of various mental disorders such as schizophrenia [15], bipolar disorder [16], unipolar depression [17], panic disorder [18], generalized anxiety disorder [19], and obsessive-compulsive disorder [20]. Hence, reducing the interval between the onset of depression and the start of appropriate treatment might improve the evolution of the depression, and even prevent progression to other medical problems.

Despite the importance of the implication of the DUI, only a few studies have investigated the factors that influence treatment contact in people with major depression [21, 22]. Altamura and colleagues [21, 22] reported that in a clinical sample of recurrent depression longer DUI was associated with an earlier age of onset and was more prevalent in females. In an Australian clinical sample, older generations were more likely to report longer delays in seeking help than younger generations [23]. To date, however, we are not aware of any studies that have investigated the socio-demographic and clinical characteristics that associate with the delay in the initial treatment contact after the first onset of lifetime depression in Japan. Nevertheless, mental health service studies in Japan are of particular interest because of the unique universal health care system of Japan in which almost everyone receives insurance. Moreover, patients have direct access to psychiatrists without having to go through primary care physicians, which may facilitate prompt contact. On the other hand, stigmatizing attitudes toward people with mental disorders are found to be more marked in Japan compared to western countries [24]. Furthermore, in Japanese cultural tradition, family members play a principal role in the decision to seek mental health services [25]. In this study, we aimed to identify the characteristics that associate with the delay in the initial treatment contact after the first onset of lifetime depression by assessing the DUI and comparing the socio-demographics and clinical characteristics in a well-characterized Japanese clinical sample.

## Methods

### Patients

We conducted a cross-sectional study involving 95 patients aged 22–64 years, undergoing outpatient depression treatment in three clinics—university hospital, general hospital, and psychiatric hospital—located in central and suburban Tokyo. The study was carried out at the depression clinic located at these three hospitals to which patients were referred for consultation and patient management by the affiliated treating psychiatrists. At their first visit, all patients provided written informed consent for being interviewed and for having the clinical information in their charts reviewed, as approved by the Ethical Committee of Keio University School of Medicine, National Hospital Organization Tokyo Medical Center, and Sakuragaoka Memorial Hospital. Only those patients who had a DSM-IV diagnosis (Diagnostic and Statistical Manual of Mental Disorders, fourth edition) of major depressive disorder based on the Structured Clinical Interview for DSM-IV (SCID) [26], were included. Exclusion criteria were past or current manic or psychotic episode, current serious and imminent suicidal intention, active substance problems, including comorbid alcohol or substance use disorders, active medical problems including major cognitive deficits, and lack of capacity to provide informed consent.

### Measures

At the first visit, all patients received comprehensive semi-structured clinical interviews and assessment battery. DSM-IV Major Depressive Disorder (MDD) with specific clinical features were assessed based on the Structured Clinical Interview for DSM-IV (SCID) [26]. Comorbid DSM-IV Axis-I disorders were diagnosed based on the Mini-International Neuropsychiatric Interview (M.I.N.I) [27]. Current severity of depression was assessed with the 17-item GRID-Hamilton Depression Rating Scale (GRID-HAMD_17_) [28]. Socio-demographics and clinical characteristics included age, gender, total education years, patient’s report of childhood abuse and bullying, family history of mood disorders, marital status [ever married (i.e., married, remarried or widowed)/never married (single)], and employment status (unemployed including medical leave/employed including homemaker and student) at the first antidepressant administration. All interviews and assessments were conducted by trained psychiatrists and clinical psychologists.

### Duration of untreated illness

DUI is defined as the time period from the onset of a patient’s first depression episode until the first antidepressant administration (treatment point) based on the definition by Dell’Osso and Altamura [14]. During the clinical interview, the first onset of lifetime depression was assessed based on the patients’ memories of their first depressive symptoms, followed by probing reference points such as critical trigger event (e.g., losing employment) and life stage during onset (e.g., “Were you already working at that department when you had these problems for the first time?”) to obtain accurate response. Methodological studies have shown that the use of references points yields more substantially plausible responses than standard procedures [29, 30]. Next, in order to assess the time of the first antidepressant administration, patients were asked to remember their first consultation with physicians or mental health professionals regarding their depressive symptoms, and whether these symptoms were linked to significant life events. Supplementary information sources such as referral letters and prescription records were also reviewed.

### Statistical analysis

To enable statistical comparisons among the DUI, we divided the duration of untreated illness into two subgroups: shorter-DUI (within 12 months) group and longer-DUI group (more than 12 months). Twelve months was chosen as a split time point based on previous studies [31, 32] where the length of the delay to seek treatment was shown to form a skewed distribution with the 1-year time point being important. Coincidently, previous DUI studies on panic disorders and general anxiety disorders [18, 19] have also used this 1-year time point to divide the DUI. Two-tailed Student’s t-tests, Mann-Whitney U-tests or Kruskal-Wallis analysis were used to compare continuous variables and Chi-square analyses or Fisher exact tests were used for categorical variables. We conducted multivariate analysis to assess the association of DUI with socio-demographics and clinical characteristics. For initial analysis, variables showing association with the shorter/longer-DUI (categorical measures) at p < 0.20 significance in the univariate analyses were entered into the backward elimination binary logistic regression model as independent variables. The dependent variable was the longer/shorter-DUI. Next, to examine the robustness of the categorical approach, we conducted a negative binomial regression analysis with the above-mentioned model by substituting DUI (in months, as a continuous measure) as dependent variable. For all other statistical tests, the two-sided significance level was set at 0.05. All statistical analyses were performed using the SPSS Version 22.0 (IBM Corp., Armonk, NY).

## Results

### Characteristics of the patients

Table 1 represents the socio-demographics and clinical characteristics of all the patients (N = 95). The mean age was 40.7 years (SD = 9.9) and more than half the patients were male. One-third of patients had current DSM-IV MDD melancholic features and approximately 30% had comorbid anxiety disorders. The mean GRID-HAMD_17_ score was 22.3 (SD = 4.0) and the mean age at the onset of the first depression was 34.0 years (SD = 11.3). Less than half the patients had never married (single) and one-fifth of them lived alone at the first treatment point. More than 60% of patients were unemployed (including medical leave) at the first treatment point. Seven patients reported history of childhood abuse (7.4%) and 21 (22.1%) reported history of being victims of bullying, while 14 (14.7%) had family history of mood disorders.

**Table 1:** **Socio-demographics and clinical characteristics of patients**

Variables	Total (n = 95)	
	n	%
Gender (Male)	57	60.0
Marital status at the first treatment point		
Ever married	56	58.9
Married and Remarried	55	57.9
Widowed	1	1.1
Never married (Single)	39	41.1
Living alone at the first treatment point	18	18.9
Working status at the first treatment point		
Employed	24	25.3
Unemployed	54	56.8
Housewife and Student	17	17.9
Subtype of DSM-IV major depressive disorder		
Melancholic features	31	32.6
Non-melancholic features (Postpartum, Atypical, no-subtype)	64	67.4
Psychiatric comorbidities		
Any Anxiety Disorders	27	28.4
Panic Disorder (including Agoraphobia)	13	13.7
Social Anxiety Dsiorder	10	10.5
Obsessive Conpulsive Disorder	5	5.3
Generalized Anxiety Disorder	1	1.1
Eating disorder	2	2.1
Patient’s-report of childhood abuse	7	7.4
Reported victims of childhood bullying	21	22.1
Family history of mood disorders	14	14.7
	**Mean**	**SD**
Age (years)	40.7	9.9
Total education (years)	15.4	1.6
Age onset of the first depression (years)	34.0	11.3
Hamilton Depression Rating Scale (Total)	22.3	4.0
	**Median [IQR 25th 75th percentile]**	
DUI; Duration of untreated illness (months)	4.0 [2.0 13.0]	

IQR: inquartile range.

### Distribution of duration of untreated illness

**Figure 1:**
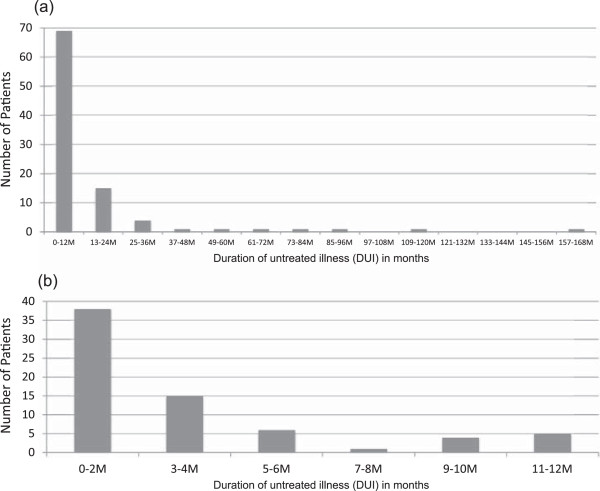
**Distribution of the duration of untreated illness. (a)** The graph illustrates the distribution of the DUI of the total sample (n = 95) stratified by 12 months. **(b)** The graph illustrates the distribution of the DUI among patients (n = 69) who sought treatment within the first year of symptom onset (i.e. DUI ≤ 12 months) stratified by 2 months.

The distribution of DUI formed a distinctly skewed J-shaped distribution (see Figure 1). Approximately 70% of patients sought treatment within the first year of depression onset and nearly 90%, by the second year. The median of DUI was 4 months (interquartile range (IQR) 25th–75th percentile, 2–13). Of patients who sought treatment within the first year of symptom onset (i.e., DUI ≤ 12 months), 55.1% (n = 38) received the first treatment within 2 months of onset. The median DUI of the shorter DUI group was 2 months (IQR, 1–4), which was significantly different from the 20.5 months median DUI (IQR, 15–44) of the longer DUI group.

When we compared DUI between the study sites, no differences were found (university hospital: 3.5 months (IQR, 2–6.8); general hospital: 6 months (IQR, 1–21.8); psychiatric hospital: 4 months (IQR, 2–13); χ^2^ = 0.56, df = 2, p = 0.76). Similarly, the depression severity as measured by GRID-HAMD_17_ were also comparable between the sites (χ^2^ = 5.08, df = 2, p = 0.08).

### Comparison of patients with longer versus shorter duration of untreated illness

Comparison of the socio-demographics and clinical characteristics between patients with longer DUI (n = 26) and shorter DUI (n = 69) is shown in Table 2. The longer DUI in patients was associated with their marital status (never married) (p = 0.01) and living status (living alone) (p = 0.02). Greater proportion of patients with longer DUI showed melancholic depression than those with shorter DUI, although not significant (n = 12 (46.2%) vs. n = 19 (27.5%); χ^2^ = 2.98, df = 1, p = 0.08). Moreover, there were no significant associations between current severity of depression and age, gender, education years, employment status, patient’s report of childhood abuse and bullying, family history of mood disorders, age at onset of the first depression, subtype of major depression, and psychiatric comorbidities.

**Table 2:** **Comparison of socio-demographics and clinical characteristics between patients with a longer/shorter duration of untreated illness**

Variables	A longer DUI (>12 months) group (n = 26)		A shorter DUI (≦ 12 months) group (n = 69)		Analysis		
	n	%	n	%	χ ^2^	df	p
Gender (Male)	16	61.5	41	59.4	0.04	1	0.85
Marital status at the first treatment point							
Never married (Single)	16	61.5	23	33.3	6.21	1	0.01
Living alone at the first treatment point	9	34.6	9	13.0	5.72	1	0.02
Working status at the first treatment point							
Unemployed	16	61.5	38	55.1	0.32	1	0.57
Subtype of depression							
DSM-IV melancholic features	12	46.2	19	27.5	2.98	1	0.08
Psychiatric comorbidities							
Any Anxiety Disorders	8	30.8	19	27.5	1.00	1	0.76
Eating disorder	1	3.8	1	1.4	0.53	1	0.48
Patient’s-report of childhood abuse	1	3.8	6	8.7	0.65	1	0.67
Reported victims of childhood bullying	6	23.1	15	21.7	0.02	1	0.89
Family history of mood disorders	4	15.4	10	14.5	0.01	1	1.00
	**Mean**	**SD**	**Mean**	**SD**	**t**	**df**	**p**
Age (years)	40.7	9.9	41.4	10.0	-0.31	93	0.76
Total education (years)	15.4	1.6	15.1	2.1	0.65	93	0.52
Age onset of the first depression (years)	34.0	11.3	36.8	10.7	-1.1	93	0.27
Hamilton Depression Rating Scale (Total)	22.2	3.9	22.3	4.0	-0.12	93	0.90
	**Median [IQR 25th 75th percentile]**		**Median [IQR 25th 75th percentile]**				p*
DUI; Duration of untreated illness (months)	20.5 [15.0 44.0]		2.0 [1.0 4.0]				<0.001

IQR: inquartile range.

*Mann- Whitney’s U-test.

It is noteworthy that the patients who never married were more likely to live alone at the first treatment point (lived alone vs. cohabited: n = 17 (43.6%) vs. n = 22 (56.4%); χ^2^ = 26.1, df = 1, p < 0.001). A more careful examination of the data among the patients who never married revealed that the DUI between those who lived alone and cohabited was not significantly different (lived alone vs. cohabited: n = 17; 13 months (IQR, 1.5–24.0) vs. n = 22; 6.5 months (IQR, 2.8–15.5); U = 184.5, p = 0.943). Similarly, when we compared the DUI between those who lived alone and cohabited among the total sample, no group difference was found (p = 0.33).

### Multivariate analysis

First, we tested a binary logistic regression model with longer/shorter DUI as the dependent variable. The independent variables were variables associated with longer/shorter DUI in univariate tests: marital status (never married), living status (living alone), and the DSM-IV melancholic features. In the final model, only marital status revealed independent association with longer DUI as compared to other variables (Table 3). This model suggests that patients with depression who are also single have an approximately 3-fold increase in odds of delaying prompt initial treatment contact (Odds ratio 3.30, 95% CI 1.27–8.58, p = 0.01). Next, to examine the robustness of the categorical approach, we conducted a negative binomial regression analysis using the same model by substituting DUI (in months, as continuous measure) as dependent variable. Marital status (never married) (χ^2^ = 8.22, p = 0.004) and the DSM-IV melancholic features (χ^2^ = 3.90, p = 0.048) were associated with DUI (months) (Table 4).

**Table 3:** **Multiple logistic regression model for delays to treatment contact (n = 95)**

Variables	B (S.E.)	Wald	Odds ratio	95% CI	p
Never married (Single) at the first treatment point	1.19	6.01	3.30	1.27-8.58	0.01
Subtype of depression (DSM-IV melancholic features)	-0.86	2.99	0.42	0.16-1.12	0.08

S.E.: standard error; CI: confidence interval.

**Table 4:** **Negative binomial regression model for delays to treatment contact (n = 95)**

Variables	B	S.E.	Wald	Wald χ ^2^	p
Never married (Single) at the first treatment point	0.88	0.31	0.28	8.22	0.004
Living alone at the first treatment point	-0.54	0.38	-1.29	1.97	0.1608
Subtype of depression (DSM-IV melancholic features)	-0.53	0.27	-1.06	3.90	0.048

S.E.: standard error; CI: confidence interval.

## Discussion

To our knowledge, this is the first study investigating the socio-demographics and clinical variables that may predict delays in initial treatment contact after the first onset of lifetime depression in Japan. There are two major findings in this study. First, the majority of patients sought treatment contact within a year of depression onset. Second, patients who failed to undergo initial treatment within a year after depression onset were more likely to be single and living alone. In our primary regression analysis, however, a marital status of “never married” showed relatively strong association with the delay in initial treatment after depression onset. This finding coincides with previous studies that suggest that marriage has a protective effect on mental health [33, 34]. Further, the DSM-IV melancholic features approached significance in the exploratory regression analysis.

Patients with depression who had never married showed a 3-fold increase in odds of delayed initial treatment contact after the first onset of lifetime depression in our sample. Several previous studies have shown the important link between marriage and treatment seeking. In a study on 121 consecutive Japanese patients who consulted a psychiatric hospital, Asai et al. [35] reported that the family members, particularly spouses, play a crucial role as motivators who decide to seek mental health care. Mohammadi et al. [36] conducted in-depth interviews of 10 Iraqi individuals to explore the process of mental health based on grounded theory, and generated a substantive theory that individuals with mental health problems deal with their stress through help-seeking, and that marriage and problem-solving skills are prominent factor within this process. Accordingly, community studies show that individuals with a higher level of social ties, which includes marriage, are more likely to use health care services [37]. Altogether these studies suggest that the increasing evidence showing shorter DUI to have a positive effect on depression treatment outcome [17] may perhaps be mediated in part by marriage, which seems to have a potential role in promoting treatment seeking.

In our multiple regression analysis, patients who had never married were associated with delayed initial treatment contact (i.e., longer DUI), but those who lived alone were not associated. In addition, no difference in the length of DUI was found between patients who lived alone and cohabited. As a possible explanation of the relatively strong association between marriage and delayed initial treatment contact, it can be hypothesized that marriage may have a higher potential in accelerating treatment contact than just cohabitation. Accordingly, in a US descriptive study, which examined the influence of sociocultural variables on patterns of help-seeking and length of delay in psychiatric patients, Asians, including Japanese, showed more persistent involvement of close family members during the treatment-seeking process, and a reluctance to accept psychiatric treatment. In contrast, Caucasians were characterized by self-initiation of help seeking and more active pursuit of treatment [38]. There is also a traditional Japanese culture view that family members, particularly spouses, of an adult with a mental disorder are held responsible for the actions of the patient, and in fact, is defined as a spouse or legal guardian in the Japanese Act on Mental Health and Welfare for Mentally Disabled (Law number: No. 123 of 1950) [39].

In the exploratory regression analysis, the DSM-IV melancholic features revealed as potential factors associated with DUI. Perhaps, this association in part may be explained by the common clinical presentation of melancholic depression, which manifests as psychomotor disturbance such as deficits in decision making and planning [40], resulting in patients delaying to seek treatment contact.

The distribution of the delay in seeking treatment after first lifetime depression onset in our study formed a J-shaped curve pattern: a distribution where the probability of initial treatment contact is the highest in the first year of symptom onset, and gradually decrease with subsequent years. This distribution pattern of delay in seeking initial treatment for depression is consistent with the epidemiological studies conducted across nations [31, 41, 42]. The median DUI was 4 months in our sample. This finding was similar to previous Japanese studies [43, 44] and reports from Italy [45] and Portugal [46], which have a similar mental health care system where patients can seek specialist care directly without previously consulting general physicians.

There are several limitations in our study. First, causality cannot be inferred from statistical associations given the cross-sectional design. Second, our sample was limited to patients treated only at the three clinical settings in Tokyo, which may cause a selection bias and hence, generalization should be made with caution. Third, although a semi-structured interview was conducted to obtain accurate responses, the exact time of onset of the first depression may not be precise since it was assessed from patient reports. Fourth, although the threshold for DUI was 12 months based on previous studies, it is arbitrary. Fifth, there is a possibility that several factors could be confounding the results, such as personality, economic status, and geographical area.

## Conclusion

Our findings suggest that most Japanese patients with depression are likely to seek treatment within 1 year of delay, and that marriage and melancholia appear to be potential predictors of the delays in the initial treatment contact after the first onset of lifetime depression. This implies that to increase prompt initial treatment contacts among people with incident episodes of major depression, it is important to not only promote screening programs using brief self-reports to detect early-onset depression [47, 48], but also improve public awareness and recognition of depression, particularly focusing on close family members. Prospective studies with larger sample sizes are required to further understand the complex relationships between socio-demographics and clinical characteristics of depression.

## Abbreviations

DUI: Duration of untreated illness
IQR: Interquartile range
DSM-IV: Diagnostic and statistical manual of mental disorders, fourth edition
SCID: Structured clinical interview for DSM-IV
MDD: Major depressive disorder
M.I.N.I: Mini-international neuropsychiatric interview
GRID-HAMD: GRID-Hamilton depression rating scale.
